# Survey of attitudes and willingness of cardiovascular specialist nurses towards nurse practitioners in Sichuan, China: A cross-sectional study

**DOI:** 10.1097/MD.0000000000046139

**Published:** 2026-05-12

**Authors:** Huaili Luo, Zhi Zeng, Dan Wen, Li Jiang, Hong Jiang, Xiaochun He, Xin Zhou

**Affiliations:** a Department of Cardiovascular Medicine, Mianyang Central Hospital, Affiliated with the School of Medicine, University of Electronic Science and Technology of China, Mianyang, Sichuan, China.

**Keywords:** attitude, cardiovascular specialist nurse, nurse practitioners, willingness

## Abstract

This study investigated the attitudes and willingness of cardiovascular specialist nurses in Sichuan, China toward nurse practitioners (NPs), analyzed the influencing factors, and provided references for further research on cardiovascular NPs. Convenience sampling was used to select 371 cardiovascular specialist nurses from 51 hospitals in Sichuan Province, China. The participants completed an online survey using a questionnaire developed for this study that assessed their attitudes and willingness toward NPs. The results indicate that cardiovascular specialist nurses hold varying attitudes toward NPs. Multiple linear regression analysis reveals that professional title and monthly income level are significant factors influencing attitude scores (*P *< .05). The primary concerns regarding the development of cardiovascular NPs include low patient understanding and acceptance, insufficient professional competence, inadequate educational preparation, and a lack of support from relevant policies and regulations for practice. Cardiovascular specialist nurses hold mixed attitudes toward NPs and still have concerns about future development prospects and potential difficulties. To address these issues, it is necessary to provide training to nurses on the required knowledge, promptly introduce policies and regulations, and establish standardized access systems and training programs to provide strong guarantees for the development of NPs.

## 1. Introduction

Cardiovascular diseases have become a major health challenge globally.^[1]^ In China, this situation is particularly severe. According to statistics, the number of people with cardiovascular diseases in China has exceeded 330 million.^[2]^ With the acceleration of population aging and the prevalence of modern, unhealthy lifestyles, the disease rate shows a continuous upward trend.^[3]^ Cardiovascular diseases, with their complex and recurrent nature, result in high rehospitalization and mortality rates, imposing a heavy burden on both patients and their families, as well as placing significant pressure on the healthcare system.^[4]^ Notably, there are only 4 practicing nurses per 1000 people in China, which is far below the average level of developed countries, and this figure is even lower in rural areas.^[5]^ This reflects the current situation of a severe shortage of nurses in primary healthcare institutions in China, and there is an urgent need to correct the problem of mismatch between the allocation of medical and health resources and the level of socioeconomic development and residents’ health needs, improve the medical service capacity of primary health teams, and thereby strengthen the effective prevention, control, and management of chronic diseases such as cardiovascular diseases.^[6]^

Nurse practitioners (NPs) are advanced nursing professionals who have completed graduate-level education and occupy the third tier in the nursing hierarchy, above specialist nurses (second tier) and registered nurses (first tier).^[7]^ NPs possess specialized knowledge and skills in health assessment, disease prevention, and health management, and are capable of making clinical decisions regarding complex issues.^[8]^ In clinical practice, NPs integrate medical knowledge and skills, with the authority to diagnose illnesses and prescribe medications, providing high-quality, low-cost, and continuous healthcare for patients with chronic diseases.^[9]^ Studies have shown that, as an innovative model for optimizing healthcare resource allocation, NPs reduce fragmentation in care. They not only significantly enhance patient experience and treatment outcomes but also demonstrate unique advantages in improving service efficiency and reducing medical costs, offering a solution to alleviate pressure on the healthcare system and address major health challenges such as cardiovascular disease.^[10]^

In countries where NPs are most well-established, such as the United States, Canada, Australia, and the United Kingdom, the professional potential of NPs in the management of chronic diseases, including cardiovascular disease, has been fully realized.^[11]^ In contrast, research on cardiovascular disease management in China has largely focused on specialist nurses or senior nurses, neglecting the professional role and unique contributions of NPs in this field.^[12]^ Although relevant studies indicate that training cardiovascular NPs is an important pathway to providing high-level care for patients with cardiovascular disease,^[13]^ the development of cardiovascular NPs in China is still in its nascent stages, with insufficient quantity and depth of related research. Given this context, a comprehensive understanding of the attitudes and willingness of cardiovascular specialist nurses toward NPs is of great significance for strengthening the future development of cardiovascular NPs and building a more robust healthcare workforce in China. To this end, this study surveyed cardiovascular specialist nurses from 51 hospitals in Sichuan Province, China, aiming to gain in-depth insights into their attitudes and willingness toward NPs, and to provide scientific evidence and practical guidance for the training and development of cardiovascular NPs.

## 2. Methods

### 2.1. Design

This study was designed and conducted as a cross-sectional study, and the reporting of this study was done following the STROBE statement. We hypothesize that these findings may help scholars identify the attitudes and willingness of cardiovascular specialist nurses toward NPs in Sichuan Province, China, and the results may provide a reliable basis for the cultivation of cardiovascular NPs in China. The specific objectives include: to understand the attitudes of cardiovascular specialist nurses toward NPs; to understand the practice willingness of cardiovascular specialist nurses toward NPs; and to explore the promoting factors and barriers for the development of cardiovascular NPs based on the attitudes and willingness of cardiovascular specialist nurses.

### 2.2. Participants

A cross-sectional online survey was conducted among cardiovascular specialist nurses from 51 tertiary hospitals in 7 cities of Sichuan Province, China, from June 1, 2024, to December 31, 2024, using convenience sampling. The inclusion criteria were: registered nurses; obtained provincial-level or above certificates for cardiovascular specialist nurses; and engaged in cardiovascular nursing work for at least 3 years. The exclusion criteria were: nurses on sick leave for more than 3 months; nurses who had left cardiovascular specialist nursing work; and nurses unwilling to participate in the survey. According to Kendall sample size calculation method, the sample size should be 5 to 10 times the number of variables in the questionnaire. This study considered 8 demographic factors and 7 scale items as independent variables. Accounting for a 20% attrition rate, the minimum required sample size was (8 + 7) × 10 × (100% + 20%) = 180 participants.^[14]^

### 2.3. Survey instruments

This study utilized a self-developed culturally specific assessment tool to evaluate the attitudes and willingness of Chinese cardiovascular specialist nurses toward NPs. First, the research team developed an initial questionnaire based on the research objectives and literature review.^[15,16]^ Subsequently, 9 nursing experts with 10 or more years of experience in the cardiovascular field were invited to conduct 2 rounds of Delphi consultation to revise the questionnaire, including 3 chief nursing supervisors and 6 associate chief nursing supervisors, with a 100% response rate for both rounds. A total of 6 experts proposed 5 suggestions for modifying 5 items. Finally, the revised questionnaire based on expert opinions was used to conduct a pilot survey among 30 cardiovascular specialist nurses. The results showed that the questionnaire completion time was 5 to 10 minutes, and after testing, the content validity of the questionnaire was 0.88, and the internal consistency reliability (Cronbach α coefficient) was 0.825, indicating that the questionnaire was valid.

The questionnaire consists of 3 parts: the first part is a general information survey form, collecting respondents’ demographic characteristics, including hospital level, age, professional title, educational background, and years of work experience. The second part investigates cardiovascular specialist nurses’ attitudes toward NPs, containing 7 items measured on a 6-point Likert scale (1 = completely disagree, 6 = completely agree), with a total score range of 7 to 42 points, where higher scores indicate more positive attitudes toward NPs. The third part examines cardiovascular specialist nurses’ willingness to practice with NPs, comprising 4 multiple-choice questions that are not quantitatively scored, aiming to systematically assess respondents’ perceptions of the expected value and potential concerns regarding NPs in clinical practice, with each item allowing multiple selections to comprehensively capture their subjective tendencies.

### 2.4. Data collection methods

Questionnaire data were collected by distributing a QR code link through the WeChat platform. After obtaining consent from each participating institution, the researchers sent the questionnaire link to WeChat groups containing heads of cardiovascular departments at various hospitals in Sichuan Province. The researchers explained the purpose, significance, and completion method of the study to these department heads, who then forwarded the link to cardiovascular nurses at their respective hospitals. Throughout the survey process, research team members promptly addressed any questions via WeChat or telephone to ensure response quality. The questionnaire did not require respondents to provide personal information such as their names. It was configured to allow only 1 submission per IP address, with all items set as mandatory questions. Respondents began completing the survey after selecting the “Agree to Participate” option.

### 2.5. Statistical analysis

Data analysis was conducted using SPSS 26.0 statistical software. For the measurement data following normal distribution, the mean ± standard deviation was used to represent them, and a *t*-test was employed for inter-group comparison. For the measurement data with skewed distribution, M (P25, P75) was used to describe them. Count data were expressed as frequency and percentage, and the chi-square test (2) was used for inter-group comparison. Multivariate logistic regression analysis was used to explore the influencing factors of cardiovascular specialist nurses’ attitudes and intentions toward NPs. A difference was considered statistically significant when *P* < .05.

### 2.6. Ethical and institutional approvals

The investigation was approved by the Ethics Committee of Mianyang Central Hospital, project number: S20240250-02. All participants were informed of the study’s purpose and assured of the confidentiality of their personal information. They were also informed of their right to withdraw from the study at any time without any consequences.

## 3. Results

### 3.1. Attitudes of cardiovascular specialist nurses toward NPs

**Figure 1.**
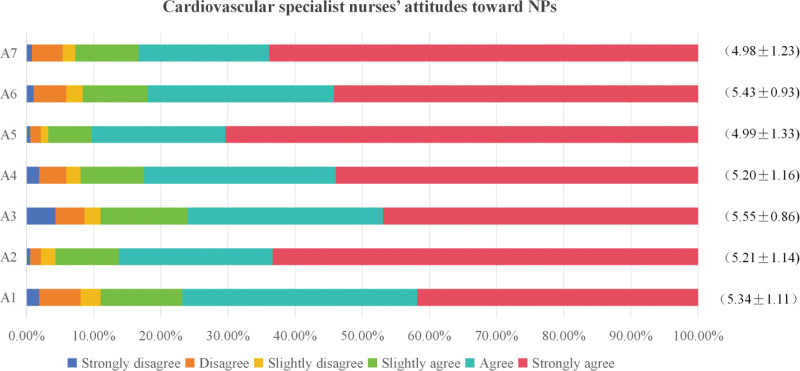
Cardiovascular specialist nurses’ attitudes toward nurse practitioners (n = 371).

A total of 412 questionnaires were collected. After eliminating invalid questionnaires, including those where all options were answered identically and those with a completion time of <5 minutes, 371 valid questionnaires were obtained (effective recovery rate of 90%). The mean score for cardiovascular specialist nurses’ attitudes toward NPs was (5.24 ± 0.88). For details on the scores of each attitude item, please refer to Figure 1.

### 3.2. Univariate analysis of cardiovascular specialist nurses’ attitudes toward NPs

The results of the univariate analysis indicate that there are statistically significant differences in the attitudes of cardiovascular specialty nurses based on different hospital levels, ages, professional titles, educational backgrounds, and monthly income levels (*P *< .05). Specific results can be found in Table 1.

**Table 1:** Univariate analysis of attitudes and willingness of cardiovascular specialty nurses toward NPs.

Item	Sort	Number (n, %)	Score ($\bar{x}\pm \;s$)	*F*	*P*
Hospital grade	First-order	2 (0.54%)	5.93 ± 0.10	4.335	.014
	Second level	69 (18.60%)	4.98 ± 1.07		
	Third level	300 (80.86%)	5.30 ± 0.82		
Gender				-0.345	.730
	Male	2 (0.54%)	5.19 ± 0.96		
	Female	69 (18.60%)	5.25 ± 0.87		
Age				9.527	<.001
	20–30 years old	93 (25.07%)	5.25 ± 0.80		
	31–40 years old	184 (49.60%)	5.08 ± 0.96		
	>41 years old	94 (25.34%)	5.55 ± 0.70		
The title of a professional post				7.183	<.001
	Junior nurse	64 (17.25%)	4.88 ± 1.10		
	Senior nurse	116 (31.27%)	5.18 ± 0.96		
	Supervisor nurse	159 (42.86%)	5.34 ± 0.73		
	Associate senior nurse and above	32 (8.63%)	5.66 ± 0.35		
Educational level				4.963	.007
	Junior college	65 (17.52%)	4.96 ± 1.12		
	Undergraduate course	297 (80.05%)	5.31 ± 0.79		
	Master’s degree or above	9 (2.43%)	4.95 ± 1.31		
Years of specialist nurses				2.743	.66
	<5 yr	70 (18.87%)	5.42 ± 0.64		
	5–10 yr	126 (33.92%)	5.12 ± 0.93		
	>10 yr	175 (47.17%)	5.26 ± 0.92		
Work roles				2.76	.065
	Outpatient nurse	42 (11.32%)	5.41 ± 0.75		
	Ward nurse	278 (74.93%)	5.18 ± 0.91		
	Head nurse	51 (13.75%)	5.44 ± 0.77		
Monthly income level				6.282	<.001
	<5000	63 (16.98%)	4.94 ± 1.08		
	5000–10,000	212 (57.14%)	5.20 ± 0.90		
	10,001–15,000	71 (19.14%)	5.49 ± 0.59		
	>15,000	25 (6.74%)	5.62 ± 0.51		

NPs = nurse practitioners.

### 3.3. Multivariate analysis of cardiovascular specialist nurses’ attitudes toward NPs

Multiple linear regression analysis was conducted with attitude score as the dependent variable and variables showing statistically significant differences in univariate analysis as independent variables. The assignment of independent variables is shown in Supplementary Material 1, Supplemental Digital Content, https://links.lww.com/MD/Q802. The results of the multiple regression analysis indicated that professional title and monthly income level were the main factors influencing attitude scores (*P* < .05), as detailed in Table 2.

**Table 2:** Multiple linear regression analysis of cardiovascular specialist nurses’ attitudes toward NPs (n = 371).

	*B*	SE	Beta	*t*	*P*	95% CI	
						Lower limit	Upper limit
(constant)	3.960	0.481		8.225	.000	3.013	4.907
Hospital grade	0.166	0.110	0.078	1.511	.132	−0.050	0.383
Gender	0.048	0.159	0.015	0.301	.764	−0.264	0.360
Age	−0.048	0.081	−0.039	−0.597	.551	−0.208	0.111
The title of a professional post	0.173	0.074	0.172	2.335	.020	0.027	0.319
Education level	0.049	0.114	0.023	0.426	.670	−0.176	0.274
Monthly income level	0.144	0.068	0.127	2.101	.036	0.009	0.279

*R*² = 0.079, adjusted *R*² = 0.061, *F* = 4.44, *P* < .001. NPs = nurse practitioners.

### 3.4. Cardiovascular specialist nurses’ willingness toward NPs

Cardiovascular specialist nurses’ willingness toward NPs is shown in Table 3.

**Table 3:** Willingness of cardiovascular specialist nurses toward NPs (n = 371).

Item	Number of respondents	Percentage (%)
Expected value of cardiovascular NPs		
Convenience for patients seeking medical care and improving patient satisfaction	349	94.07%
Improving doctors’ work efficiency	343	92.45%
Enhancing the quality of medical and nursing services	338	91.11%
Broadening career development paths for nurses	325	87.60%
Promoting the development of nursing science	312	84.10%
Value of becoming cardiovascular NPs for personal development		
Differentiating from traditional nursing roles and enhancing nurses’ image in patients’ minds	353	95.15%
Stronger independence for nurses, reducing dependence on doctors	347	93.53%
Expanding nursing functions and promoting balance of medical resources	345	92.99%
Improved status of nurses, leading to higher activation in nursing work	327	88.14%
Concerns about becoming cardiovascular NPs		
Low understanding and acceptance by patients	334	84.64%
Insufficient professional competence and lack of education preparation	281	75.74%
Lack of protection and support from relevant policies and regulations for practice	258	69.54%
Unclear professional boundaries and scope of practice	257	69.27%
Conflict with doctor roles	228	61.46%
Practice not included in the medical insurance system	216	58.22%

NPs = nurse practitioners.

## 4. Discussion

### 4.1. Cardiovascular specialist nurses show conditional interest in NPs, but there are significant barriers to implementation

The findings of this study show that cardiovascular specialist nurses’ attitudes and willingness toward NPs are characterized by conditional interest with significant implementation barriers. This is consistent with research results on nurses’ attitudes and willingness toward NPs in other countries.^[17]^ On one hand, cardiovascular specialist nurses acknowledge the value of NPs, believing that the development of this role can benefit patients, physicians, and nurses alike. It can enhance patient satisfaction, improve physicians’ work efficiency and the quality of healthcare services, elevate nurses’ image in the eyes of patients, and reduce the sense of dependence on physicians. International reports have already confirmed that NPs play an important role in many aspects, such as helping to identify previously unknown cardiovascular risk factors through systematic screening and comprehensive management, accurately calculating the Framingham Risk Score, and assisting in setting cardiac health priorities and personalized treatment goals.^[18]^ On the other hand, cardiovascular specialist nurses also have some concerns regarding NPs. These concerns mainly arise from 3 areas: patients, national policies, and physicians. The cardiovascular specialist nurses surveyed in this study indicated that the primary concerns about becoming cardiovascular NPs include low patient understanding and acceptance; insufficient professional competence, lack of educational preparation, and the absence of protection and support from relevant policies and regulations in practice. These concerns are largely consistent with the challenges faced by other countries in developing NPs^[19]^ and are also in line with the findings of related exploratory studies on NPs in China.^[20]^

1. Patient acceptance and support are key factors in promoting the implementation of cardiovascular NPs.^[21]^ However, the role of NPs in China’s current healthcare system is still in its early stages of development, and patients have limited awareness of their scope of practice and professional capabilities, which may lead to insufficient trust and consequently affect the effectiveness of nurse–patient communication.^[22]^ Additionally, patients are generally accustomed to the traditional physician-led diagnosis and treatment model and still have reservations about the independent diagnosis and treatment services provided by NPs.^[20]^ Therefore, it is necessary to actively promote the NP role through multiple channels, create a social atmosphere that supports their development, and enhance public recognition. At the same time, public education should be strengthened to systematically popularize the professional value and service scope of NPs, clarify their role positioning and collaborative mechanisms within the healthcare team, and strive to build a professional and trustworthy nursing image, thereby effectively enhancing patients’ awareness and trust in NPs and promoting the development of NPs in the cardiovascular field in China.^[23]^
2. Professional competence is the foundation for implementing cardiovascular NPs, but there is still room for improvement in China regarding the competency development and standardized management of cardiovascular NPs. To address this, it is recommended to draw from Australia’s competency-based education model and develop comprehensive competency standards that cover multiple dimensions such as cardiovascular disease assessment, management, intervention, and health education, tailored to the characteristics of cardiovascular specialties, in order to provide clear guidance for education and training.^[24]^ Meanwhile, it is necessary to introduce the Licensure, Accreditation, Certification, and Education regulatory consensus model as a framework to systematically standardize the cardiovascular NP training system.^[20]^ Through its unified accreditation bodies and enhanced regulatory mechanisms, this approach can enhance the professionalism and academic rigor of nursing practice, thereby laying a solid talent foundation for the standardized development of cardiovascular NPs.
3. Sound legal protection is essential for the professional development and rights protection of cardiovascular NPs. Countries such as the United States have pioneered the legalization of NP practice, with their scope of practice expanding to multiple areas including mental health,^[25]^ oncology,^[26]^ and emergency care,^[27]^ becoming an important component of the healthcare system. Subsequently, many countries have explicitly granted NPs independent practice rights through legislation,^[28]^ and while expanding their practice authority, they have actively explored the establishment of scientific and effective regulatory mechanisms to achieve balanced development between practice autonomy and standardized practice.^[29]^ However, China currently has not yet established a comprehensive legal framework and policy support system for NPs practice, which imposes numerous limitations and uncertainties on NPs in clinical practice. In the future, it is necessary to draw upon the developmental experiences of other countries,^[30]^ promote funding support and pilot implementation of innovative models through extensive dialogue and collaboration with various stakeholders, gradually expand nurses’ prescribing authority, and lay the foundation for the legal development of cardiovascular NPs in China. Furthermore, theoretical frameworks can be integrated into the implementation process of cardiovascular NPs; these frameworks guide the formulation and optimization of practical strategies by identifying barriers and facilitators present in different environments and contexts, thereby promoting the scientific basis and adaptability of the cardiovascular NP system construction.^[31]^

### 4.2. Influencing factors of cardiovascular specialist nurses’ attitudes toward NPs

The multiple linear regression analysis of cardiovascular specialist nurses’ attitudes toward NPs shows that professional title and salary level are important factors influencing their attitudes. In terms of professional title, the analysis of this study indicates that cardiovascular specialist nurses with higher professional titles hold more positive attitudes toward NPs. This may be because nurses with senior titles have established stable professional status and authority and are less likely to view NPs as a threat. At the same time, they are more concerned with the overall optimization of the healthcare system and can objectively recognize the value of NPs in patient health services. Similar to the research findings of Minhui Dai et al,^[32]^ that study also found that healthcare professionals with senior titles had significantly higher acceptance of emerging healthcare roles than those with junior titles. It is recommended to invite senior-title nurses to participate in NP-related policy-making and training design, leveraging their leadership role, while providing career planning guidance for nurses with middle and low titles to enhance their understanding and identification with the NPs role.^[33]^ In terms of salary level, the analysis of this study indicates that cardiovascular specialist nurses with higher monthly income levels hold more positive attitudes toward NPs. This may be because nurses with higher incomes have a solid economic foundation and strong market competitiveness, with a higher tolerance for career transition risks, enabling them to view the career development opportunities brought by NPs more rationally. Similar to the research findings of AlSadah et al,^[34]^ that study confirmed that economic security is an important predictor for healthcare professionals’ acceptance of new roles. It is recommended to provide advanced training and international exchange opportunities for high-income nurses to broaden their professional horizons; for low-income nurses, establish special support funds and entrepreneurial guidance to reduce the economic barriers to transitioning to NPs,^[35]^ thereby promoting positive acceptance of the NP role among nurses at different income levels.

### 4.3. Limitations

This study has the following limitations: First, the small sample size limits the representativeness and statistical power of the research findings. Due to the limited number of cardiovascular specialist nurses participating in the survey, it may not comprehensively reflect the attitudes and willingness toward NPs among nurses with different backgrounds, which affects the generalizability of the research conclusions. Second, the study was confined to a single geographical region (Sichuan Province, China). However, differences in medical policies, resource allocation, and nursing practices across regions may result in the research findings not applying to cardiovascular specialist nurse populations in other areas. Future research should expand the geographical coverage and include multiregion samples to improve the external validity of the study. Finally, the study used self-structured instruments to collect data. Although this method facilitates questionnaire design for specific research questions, it may have issues with insufficient instrument validity and reliability, and the responses from cardiovascular specialist nurses may be influenced by personal understanding and social desirability bias. It is recommended that future research adopt mixed methods, integrating quantitative and qualitative data collection techniques to enhance the comprehensiveness, depth, and reliability of the research findings through multi-angle and multilevel data triangulation.

## 5. Conclusion

Cardiovascular specialist nurses hold mixed attitudes toward NPs and still have concerns about future development prospects and potential difficulties. Although the development of cardiovascular NPs can not only optimize medical resource allocation and enhance the management efficiency of cardiovascular diseases but also effectively reduce disease risks through professional screening and personalized interventions, create broader career development opportunities for nursing personnel, and help cultivate leading talents in the nursing field. However, it also faces practical challenges such as unclear scope of practice, inadequate laws and regulations, immature professional training systems, and insufficient social recognition. In the future, countries should formulate appropriate NPs development strategies based on the characteristics and development stages of their own healthcare systems.

## Acknowledgments

The authors are very grateful to all the cardiovascular specialist nurses who participated in this study.

## Author contributions

**Conceptualization**: Huaili Luo, Zhi Zeng.

**Data curation**: Dan Wen, Li Jiang, Hong Jiang, Xiaochun He.

**Writing – original draft**: Huaili Luo, Zhi Zeng.

**Writing – review & editing**: Xin Zhou.

## Supplementary Material
