# Supplementary material for: Survey of attitudes and willingness of cardiovascular specialist nurses towards nurse practitioners in Sichuan, China: A cross-sectional study
Source: Medicine (Baltimore). 2026 May 12;104(49):e46139. doi: 10.1097/MD.0000000000046139 (PMC12688874; doi:10.1097/MD.0000000000046139)
Supplement: Supplementary file 1 [file medi-104-e46139-s001.docx]

Supplementary Material 1 KAP Questionnaire for Glucose Management

| ****Variable**** | ****Coding**** |
| --- | --- |
| Hospital level | 1 = Primary hospital, 2 = Secondary hospital, 3 = Tertiary hospital |
| Gender | 1 = Male, 2 = Female |
| Age | 1 = 20–30 years, 2 = 31–40 years, 3 = >41 years |
| Professional title | 1 = Nurse, 2 = Senior Nurse, 3 = Nurse-in-charge, 4 = Associate Chief Nurse or above |
| Education level | 1 = Junior college, 2 = Bachelor, 3 = Master or above |
| Monthly income level | 1 = <5000 RMB, 2 = 5000–10000 RMB, 3 = 10001–15000 RMB, 4 = >15000 RMB |
